# The Fortunes of Genomic Medicine: A Quarter Century of Promise

**DOI:** 10.1111/ahg.70011

**Published:** 2025-07-17

**Authors:** Steve Sturdy

**Affiliations:** ^1^ The University of Edinburgh Edinburgh UK

**Keywords:** economic policy, genomic medicine, national health policy, sociology of medicine

## Abstract

**Background:**

The culmination of the Human Genome Project in the early 2000s came wreathed in promises of a revolution in medicine and healthcare. The ensuing quarter century has seen remarkable growth in genomic medicine, as well as notable shifts in the promissory rhetoric that accompanies it.

**Methods:**

This essay draws on a contextualist close reading of scientific and policy literature published from the 1990s to the present, using thematic and narrative analysis informed by perspectives from the sociology of expectations, to examine the role of different kinds of promissory claims in shaping the development of genomic medicine in the UK, and particularly England, over that period.

**Results:**

Early promises about the medical benefits that will follow from the development of genomic medicine have been scaled back and refocused as the possibilities and limitations of genomic technologies have become apparent; while investment has shifted from basic discovery resources aimed at elucidating the genetics of common disorders to clinical facilities focused on the genomics of rare diseases and cancer. Research in these areas has delivered a range of highly‐publicised diagnostic and therapeutic innovations, but so far the benefits to patients have generally been modest or confined to relatively small populations, and come at a high cost, both financial and human. Meanwhile, a rather different set of promises, focused on economic growth through biomedical innovation, has been instrumental in shaping how the field of genomic medicine has developed, especially within the National Health Service. One consequence has been a blurring of the distinction between medical care and biomedical research, with genomic medicine patients and their data increasingly being reframed as an economic resource for purposes of commercially‐driven innovation.

**Conclusion:**

In this context, efforts to persuade patients of the personal or public value of research participation, and especially proposals to abandon the principle of clinical non‐directiveness in genomic healthcare, raise uncomfortable questions about just whose interests genomic medicine, as currently constituted, is best designed to serve.

## 1 Introduction

As the Human Genome Project came to its culmination in the announcement of increasingly complete draft reference sequences between 2000 and 2004 (International Human Genome Sequencing Consortium 2001, 2004; see also Nurk et al. 2022), the chorus of promissory claims about the future of medicine, which had accompanied the Project from its inception, reached a crescendo. No less a figure than US President Bill Clinton, speaking at a press conference to announce the completion of the first draft, declared that ‘Genome science … will revolutionize the diagnosis, prevention, and treatment of most, if not all, human diseases’, while UK Prime Minister Tony Blair, joining by satellite link, concurred: ‘let us be in no doubt about what we are witnessing today: a revolution in medical science whose implications far surpass even the discovery of antibiotics’ (White House, Office of the Press Secretary 2000).

In this essay, I review the development of genomic medicine over the intervening quarter century to consider what has become of that promise. I do so not in a spirit of accounting – I am not interested in applauding those predictions that turned out to be correct, nor in demeaning those that failed. Rather, I take my lead from work in the field of ‘the sociology of expectations’, which examines the role that anticipatory claims about what might be achieved in the future play in shaping scientific activity in the present (Brown and Michael 2003; Borup et al. 2006; Hedgecoe 2004). A promise is not just a prediction; it is a commitment, which serves to orient and align collective action, garner wider support and channel the flow of money and other resources. By viewing the development of genomic medicine in the light of the promises that attended it, my aim is to illuminate the different kinds of aims and expectations that have shaped the remarkable field of medical activity that we now know by that name. In that respect, I am interested as much in how the promise of genomic medicine has changed over time, in response to changing circumstances, achievements and disappointments, as in whether or not particular promises were ever fulfilled.

My focus in this essay is on the United Kingdom, especially England. Many of the points I make will also apply, in broad terms and with suitable adjustments and qualifications, to other national and international contexts. But my aim is to tease out how specific scientific, medical and social factors have interacted to shape genomic medicine to its current form – and for that purpose it is best, at least for now, to concentrate on one particular national case. I start by looking at how changing scientific and medical promises, about what kind of knowledge genomics might generate about health and illness and how medical practice should be transformed as a result, have shaped the kind of services around which the present NHS Genomic Medicine Service is configured. I next turn to document a rather different aspect of genomic promise, namely the expectation that the development of new genomic technologies could help to drive industrial innovation and economic growth, particularly in the healthcare sector; this line of promise, in particular, was instrumental in driving and shaping the growth of genomic medicine. Finally, I consider what these developments mean for the patients and research participants who pass through the evolving institutions and practice of genomic medicine and how the different promises in play may or may not speak to their values and interests.

## 2 The Promise of the Human Genome Project and the Disappointment of GWAS

With the wisdom of hindsight, it would be easy to dismiss the revolutionary promises that attended the completion of the Human Genome Project as over‐excited hype. At the time, however, those promises were rooted in deeply held hopes for what genomics, especially DNA sequencing, would make possible. Those hopes dated back at least to the beginnings of the Human Genome Project in the late 1980s and rested on expectations about how knowledge of the human genome sequence would make it possible to unravel the genetic determinants of the most prevalent forms of human illness.

It had long been known that common disorders such as coronary heart disease and cancer often come with a significant degree of inherited risk. But the sources of that risk were hard to pin down. In the case of rare single‐gene disorders such as cystic fibrosis, the causal genetic variants could be tracked with relative ease through successive generations of affected families, and by the early 1990s, molecular geneticists were beginning to map, clone and sequence the genes associated with a growing number of such disorders. By contrast, common disorders like heart disease were assumed to be influenced by multiple genetic risk factors, making it much harder to trace their transmission from one generation to the next. By the end of the 1990s, a handful of genetic variants had been found to be associated with seriously elevated risk of certain strongly familial forms of cancer, heart disease and dementia. But it was widely assumed that the vast majority of common disease was attributable to genetic variants with rather smaller effect size. The decision to invest in a major project to sequence the entire human genome was motivated by the expectation that it would lead to new, more powerful methods of identifying the elusive genetic risk factors (Sturdy 2020).

Researchers also had clear expectations about what those methods might involve. They had assumed for some time that it should in principle be possible to adapt standard techniques from the field of risk factor epidemiology, comparing affected cases with unaffected controls to identify statistical associations between particular genomic variants and the occurrence of particular kinds of illness. Attempts to apply such ‘case–control’ methods in the 1980s and early 1990s had failed because the available maps of human genomic variants were insufficiently fine‐grained, and the rates of genetic recombination were too high, to yield consistent associations (Lander and Schork 1994). The Human Genome Project, and the effort to generate a complete reference sequence of the human genome, was intended to address that problem by providing a baseline against which genomic variants could be identified. But it was only one of a number of initiatives that were all intended to contribute to the assembly of much more detailed and fine‐grained maps of different kinds of variation. By mid‐2000, an international consortium of public and commercial research organisations had come together to fund what would quickly become the definitive database of single‐nucleotide polymorphisms (National Human Genome Research Institute 2000), while in 2002, the International HapMap Project began collecting and analysing samples from four populations of supposedly distinct geographical descent to map chromosomal patterns of variation and co‐inheritance (International HapMap Consortium 2003; National Human Genome Research Institute 2002; Reardon 2017).

Meanwhile, the next steps were being taken towards finding the genetic determinants of common disorders. Case–control methods of identifying disease risk, whether it be environmental or genetic, depend on documenting and comparing sufficiently large populations of well‐characterised cases and controls; the larger the population, the more sensitive the analysis. The public and private biobanks established in countries around the world from the late 1990s onwards were intended to satisfy that need by collecting the genomic sequence data of large numbers of individuals and bringing them together with their detailed personal and clinical data.

British funders and researchers had made a major contribution to the Human Genome Project, and they were keen to remain at the forefront of efforts to bring genomics to bear on medicine. The possibility of establishing a national biobank was already under discussion by 1999, and UK Biobank began recruiting its first participants in 2005, with full recruitment beginning in 2007. At the same time, as the means for identifying the sought‐for genetic risk factors began to take shape, the associated promises also became more clearly articulated, not least in government policy documents (e.g. Department of Health 2003, 2008; House of Lords, Science, and Technology Committee 2009). A number of gene variants had already been identified which affect how individuals respond to certain drugs, and further work in this area was widely expected to result in routine use of pharmacogenomic testing and prescribing (e.g. Department of Health 2008; Hedgecoe 2004; Hedgecoe and Martin 2003). More ambitiously, scientists predicted that research into the role of different genomic variants in the aetiology of common disorders would make it possible to target therapies more precisely at the particular pathological processes in play, while testing patients to determine just what variants they possessed would ensure they were prescribed the most effective treatment – an approach that came to be known as ‘stratified medicine’ (House of Lords, Science, and Technology Committee 2009). And even more ambitious yet, it was widely hoped that the such knowledge would lead to the development of new therapeutic interventions, designed to target the pathological processes in which those genes were implicated (House of Lords, Science, and Technology Committee 2009; Tutton 2014).

In parallel with the establishment of biobanks, researchers in several countries launched proof‐of‐principle initiatives, using other sources of personal medical and genomic data, with the aim of determining whether case–control methods were actually capable of delivering meaningful information about genetic risk factors. Methods of conducting case–control analysis had been progressively refined to work with genomic data, particularly using what came to be known as genome‐wide association studies (GWAS) (Heeney 2021). One such was the Wellcome Trust Case‐Control Consortium (WTCCC), launched in early 2005, which brought together members of over 50 UK‐based research groups to compare cases of seven different common disorders with controls drawn from a long‐established UK birth cohort study and from the British blood donation service. The results, published in June 2007, were distinctly double‐edged. On the positive side, the WTCCC provided compelling affirmation that case–control methods could indeed identify genetic variants associated with elevated risk of certain common diseases. The researchers successfully identified 24 genomic loci that were strongly associated with increased risk of one or another of the diseases being studied. They concluded that ‘GWA analysis provides a highly effective approach for exploring the genetic underpinnings of common familial diseases’ (Wellcome Trust Case Control Consortium 2007, 673). Along with a number of other studies, this achievement was seen as sufficiently important for the magazine *Science* to hail GWAS as the ‘breakthrough of the year’ (Pennisi 2007). On the negative side, while the associations between the novel genomic loci and the risk of different diseases were statistically robust, the size of that risk was disappointingly small. ‘The novel variants we have uncovered are characterized by modest effect size’, cautioned the authors of the WTCC report, ‘and even these estimates are likely to be inflated’ (Wellcome Trust Case Control Consortium 2007, 674). For all that the findings provided satisfying proof of methodological principle, from a medical point of view they offered ‘limited potential … (singly or in combination) to provide clinically useful prediction of disease’ (675).

Faced with this and other similarly disappointing GWAS results, researchers’ initial response was to try to salvage the situation by increasing the power of their methods. Like other early GWAS projects, the WTCCC had hypothesised that the risk of common disorders was due to a relatively small number of genetic variants, each of which occurred relatively frequently in the population – the so‐called common disorder–common variant hypothesis. The WTCCC had accordingly been designed to detect risk variants occurring with a population frequency of 5% or more. Researchers now speculated that perhaps that risk was actually due to a larger number of variants, each of which was individually rarer than anticipated. Various studies were therefore launched to test that hypothesis, using more detailed genome maps to enable more powerful statistical analyses. One such was the UK10K Consortium, launched in 2010, which adopted the latest sequencing technology to examine the coding regions of the genome at a much higher resolution than the WTCCC, in order to ‘substantially increase the study's power to identify rare variants’ linked to a number of common disorders (UK10K Consortium 2011). The results only compounded earlier disappointments, identifying new disease associations, but none of a sufficient size to be medically actionable. Reviewing their findings in 2015, the researchers concluded that ‘In contrast to previous projections, from this analysis of a wide range of biomedical traits there was no evidence of low‐frequency alleles with large effects upon traits’ (UK10K Consortium 2015, 84). Like the WTCCC before it, the Project had provided ‘proof‐of‐principle evidence on the value of the large‐scale sequencing data for complex traits’. But it also indicated that ‘there are few low‐frequency large effect “quick wins” that make substantial contributions to population trait variation and that can be discovered from sequencing studies of few thousands individuals’ (87).

These and other studies effectively ended researchers’ hopes that GWAS would lead to the identification of genetic variants that made a major contribution to the risk of developing common disorders. Some researchers have sought to rescue the clinical relevance of GWAS by combining multiple small genetic risk factors to generate actionable polygenic risk scores (e.g. Torkamani et al. 2018) – though at present, the dominant view is that the medical value of such scores remains low (Hingorani et al. 2023). More typically, advocates of GWAS have responded by playing down earlier predictions of medical revolution and stressing instead the scientific advances under way (Heeney 2021), reframing GWAS as a means to ‘better understand the biology of disease’ – albeit while still holding onto the hope that such understanding ‘will lead to prevention or better treatment’, particularly if GWAS can be augmented with ‘new types of data, new molecular technologies, and new analytical methods’ (Visscher et al. 2017, 5). GWAS are also increasingly used in drug discovery to identify potential gene products and pathways to target, and to provide supporting evidence in drug approval applications (Ochoa et al. 2022; Rusina et al. 2023). The UK government accordingly continues to promote the development of UK Biobank alongside new clinical data sources and innovations in big data analytics, in the expectation that this evolving big‐data ecosystem will help to foster ‘the next generation of diagnostics and clinical tools—including the evaluation of polygenic risk scores (PRS), drug discovery, and smart clinical trials’ (HM Government 2021).

## 3 Back to the Clinic – Rare Diseases and Cancer

Meanwhile, as hopes that GWAS would lead to a transformation in medical practice waxed and then waned, other less‐publicised lines of research in genomic medicine continued to develop and would eventually replace GWAS as the principal bearers of genomic promise. One was the field of rare genetic disorders. With the launch of the Human Genome Project, the spotlight of attention had shifted away from rare disorders, in the expectation that the genetics of common disorders would soon be unravelled. But clinical geneticists had continued to collaborate with molecular geneticists to elucidate the biological processes underlying a growing range of rare diseases, and the rapid development of sequencing technologies and detailed genome maps that accompanied the Human Genome Project further accelerated this progress. As a result, the early 2000s saw continuing expansion, both in the range of conditions for which genetic causes were identified and in the numbers of pathogenic variants identified for each condition (García‐Sancho et al. 2022).

The other medical domain in which the new genomic technologies were helping to deliver new knowledge of disease was oncology, particularly the study of tumours. Here too, clinicians and molecular geneticists had been working together since the 1980s to elucidate the role of genomic variants, both inherited and somatic, in tumorigenesis, using both family methods and direct analysis of tumour genomes to identify relevant variants (Sturdy 2021), and here too, the technological and methodological developments associated with the Human Genome Project had helped to accelerate that research. By the early 2000s, the idea that cancer is fundamentally a genomic disorder, and that knowledge of genetic variation and the cellular processes in which it was implicated offered the best hope of effective, targeted treatments, was becoming widely accepted in medical and scientific circles.

Scientific advances in both these fields of medicine were repeatedly invoked to support the broader promissory claims that attended the completion of the Human Genome Project, as well as the investment and policy support that such claims helped to mobilise. Screening for cystic fibrosis and sickle cell disease, for instance, was a well‐rehearsed example of progress from the field of rare diseases (House of Lords, Science, and Technology Committee 2009, 39), while the approval in 2001 of Gleevec, a novel therapy targeting the variant gene product responsible for chronic myeloid leukaemia, served as a poster child for cancer genomics (100). But so long as attention focused on GWAS and the promise of solving the problems of common disorders, researchers studying the genomics of rare diseases and cancer tended to be left to follow their own interests. Additional investment in NHS clinical genetics centres was announced in 2003, for instance, but it was intended primarily to ‘boost capacity’ and ‘enable better handling of work and communications within and between participating laboratories’ to meet what policy makers anticipated would be the expansion of genetic testing resulting from the implementation of stratified medicine – a matter of downstream application of discoveries made elsewhere, in UK Biobank and six new genetics knowledge parks, rather than a turn to clinical genetics as a site of research in its own right (Department of Health 2003).

By the early 2010s, however, researchers and clinicians studying the genomics of rare diseases and cancer increasingly found themselves in the policy spotlight, as official enthusiasm shifted towards the clinic as the most promising site for effecting a genomic transformation in medicine. In December 2012, the UK Government announced the launch of what would come to be known as the 100,000 Genomes Project (100KGP). The Project marked a significant shift in the direction of genomic medicine policy and promise. Where attention had previously focused primarily on developing basic discovery resources such as UK Biobank, the 100KGP was concerned with building clinical genomics capacity – and, in particular, with enabling clinicians to access whole‐genome sequencing if they thought it might throw light on a patient's condition. With an initial funding allocation of £100 million, as well as substantial contributions from the Medical Research Council and the Wellcome Trust and significant buy‐in from industry, the project was intended to ‘train a new generation of British genetic scientists … train the wider healthcare community in harnessing this technology … pump‐prime DNA sequencing for cancer and rare inherited diseases … [and] build the NHS data infrastructure to ensure that this new technology leads to better care for patients’ (HM Government 2012a).

The promissory rhetoric that surrounded the launch and roll‐out of the 100KGP was also different in tone and focus from what had gone before. Talk of common disorders was largely absent. Claims that the Project had ‘the potential to transform the future of healthcare’ persisted but were now confined specifically to ‘diagnosis and treatment for patients with cancer and rare diseases’ (NHS England 2014). If the scope of the promised genomic transformation medicine had narrowed, however, its arrival was seen to be much more imminent. Where the medical promise of GWAS had usually been projected into some unspecified future, the 100KGP came with ‘a clear goal of accelerating the findings from the programme back into mainstream healthcare at the fastest possible pace, meaning more rapid results for patients’ (NHS England 2014). Plans included the creation of a network of regional Genomic Medicine Centres which would sequence and analyse genome samples sent in by oncologists and clinical geneticists with a view both to generating new knowledge of disease and to feeding back such findings as might prove useful in clinical practice. From a laboratory‐led revolution in the understanding of common disorders, the promise of genomic medicine had now been redefined around a rolling programme of innovation in the care of people with rare diseases and cancer.

Initially, claims about the promise of the 100KGP tended to foreground cancer rather than rare diseases, citing hopes for ‘life‐saving new drugs, treatments and scientific breakthroughs, which experts predict could significantly reduce the number of premature deaths from cancer within a generation’ (HM Government 2012b). This promise has proved difficult to deliver at any scale. With the introduction of new genomic technologies through the 100KGP and, from 2018, the NHS‐wide Genomic Medicine Service, oncology has developed into a remarkably sophisticated research enterprise, recruiting a growing proportion of patients into increasingly complex clinical trials and bringing together multi‐disciplinary teams of clinical oncologists, molecular geneticists, bioinformaticians and others to monitor and analyse the effects of a growing range of new genomically targeted interventions (Day et al. 2016; Kerr et al. 2021). As a result, the practice of oncology has been transformed into a distinctly novel form of ‘experimental care’ (Cambrosio et al. 2018; Day et al. 2021), in which the distinctions between research and care have largely disappeared. While this new clinical endeavour has yielded abundant insights into carcinogenesis and the biological complexities of tumours, the clinical pay‐offs appear decidedly modest. New genomic technologies have led to significantly improved outcomes for some specific forms of cancer – CAR‐T cell therapy for certain kinds of blood cancer, for instance. But the available evidence suggests that, overall, while clinical genomics has enabled much more targeted treatment of a range different tumour types (Sosinsky et al. 2024), the impact on treatment outcomes has been limited (Prasad et al. 2017; Schnog et al. 2021; Shendure et al. 2019).

Compared to cancer, rare diseases received far less attention in the run‐up to the 100KGP. By 2017, however, the bridesmaid was coming to outshine the oncological bride. With planning already under way for what would soon become the Genomic Medicine Service, the Chief Medical Officer for England devoted her annual report for 2016 to genomic medicine, extolling the achievements to date and adumbrating the benefits to come (Davies 2017). Among those achievements, she noted especially the Deciphering Developmental Disorders (DDD) Study – a joint initiative between the Wellcome Sanger Institute and the NHS genetic services which had launched in 2011 (Firth and Wright 2011). By 2016, the Study had undertaken detailed genomic analysis of 12,000 children with undiagnosed severe developmental disorders and delivered genetic diagnoses for around 40% of them, including more than 30 previously unknown genetic conditions. Rolling out these kinds of diagnostic facilities across the entire NHS would go a long way to end the ‘so called “diagnostic odyssey”’ that confronts so many patients and their families, promised the CMO (Davies 2017, 9). Subsequent developments, both in the United Kingdom and elsewhere, have continued to affirm the power of whole genome sequencing to deliver genomic diagnoses in a substantial proportion of suspected rare disease cases (e.g. Turro et al. 2020; Wigby et al. 2024).

Stories of diagnostic puzzles solved made for good publicity for the nascent Genomic Medicine Service. But while enhanced diagnostic capabilities have enabled a growing number of rare disease patients and families to access existing forms of medical and other kinds of care and assistance, the impact on patient outcomes, as with cancer, has been limited. Recent years have seen some much‐publicised therapeutic innovations for a handful of rare diseases, including molecular technologies that act at the level of the genome itself – so‐called ‘gene therapies’. But for the great majority of rare diseases and rare disease patients, measurable improvement in therapeutic outcomes remains more a matter of promise than actuality (Shendure et al. 2019; Weymann et al. 2024; Wigby et al. 2024). Nonetheless, that promise has been sufficient for rare diseases to be given an increasingly prominent place in government plans for rolling out genomic medicine: from a rather thinly specified ‘strategy’ in 2013 to a formal ‘action plan’ in 2022 (Department of Health and Social Care 2018, 2021, 2022a; see also Rare Disease Research Landscape Steering Group 2023).

All of this comes at a price, however – not just the financial cost of providing the technology and infrastructure that is central to the work of genomic medicine but also the human costs involved in implementing new, technically and organisationally onerous forms of practice at speed and under pressure (Samuel and Farsides 2017). Patients pay too, in the form of what sociologists call the ‘clinical labour’ involved in taking part in medical research (Cooper and Waldby 2014): the time and effort of attending clinics and providing samples and data; the physical strain of undergoing experimental treatments and enduring side‐effects; and the emotional labour of dealing with hope and disappointment for oneself and one's family and friends (Dheensa et al. 2019; Mitchell 2010). And the costs are likely to continue to increase, particularly as new medicines come on stream for both cancer and rare diseases. In the field of cancer, the costs of novel therapies have escalated out of proportion with the observable benefits they deliver to patients (Saluja et al. 2018: Sullivan 2024), while in both cancer and rare diseases, the usual cost‐effectiveness limit on what drugs can be provided through the NHS has been raised substantially, with additional funding set aside for prescribing novel medicines even before they are fully assessed for value (Department of Health and Social Care 2022c; National Institute for Health and Care Excellence 2024, 2022; NHS England 2016). Seen from a health economics perspective, genomic medicine looks like a very costly way to secure what, at a population level, are still only rather marginal gains in public health.

## 4 Economic Promise

Seen from another perspective, however, the expense is actually the point. The UK Government's efforts to promote genomic medicine have never been solely about delivering medical benefits. From the start, they were also shaped by expectations of economic benefit, which have been at least as potent in driving policy and mobilising resources as the promises of medical transformation that tended to make the headlines. Economic promise had attended and helped to drive the development of genomics from at least as far back as the start of the Human Genome Project (Cook‐Deegan 1994). But the successful delivery of the first drafts of the human genome sequence in the early years of the new millennium occurred at a particularly opportune moment in the development of British economic policy.

Under the New Labour government elected in 1997, the idea that strategic public investment in research and innovation could stimulate economic growth became a fundamental tenet of government policy. Biomedical innovation was a particular focus. The United Kingdom was still home to a vibrant pharmaceutical industry, sustained in part by a strong public research base in the universities. It also had the National Health Service – the largest single, integrated healthcare system in the world, and a site of scientific and medical innovation in its own right. But policy makers and pharmaceutical industry leaders urged that much more could be achieved if these different elements were brought into closer alignment and engagement with one another. But it was the NHS, seen as ‘a distinctive asset whose potential has yet to be realised’ (Department of Trade and Industry et al. 2003, 13), that received the most attention. ‘The health services sector is so large, it should become a vibrant sector of the economy, providing not only a healthy population and workforce, but also itself contributing to employment and national wealth’, declared an influential 2002 review of health expenditure (Wanless 2002, i).

Starting in the early 2000s, under the mantra of ‘health and wealth’, the government launched a series of policy initiatives that aimed to open up the NHS to both public and commercial research, promising that this would bring not just new and better treatments for patients but also economic benefits (Department of Health and Association of the British Pharmaceutical Industry 2001; Department of Trade and Industry et al. 2003; Department of Health and Social Care 2006; Cooksey 2006; Atkinson et al. 2019). A key aim was to make the United Kingdom a leading competitor in the international market for industry‐sponsored clinical research. UK‐based pharmaceutical companies were among those expected to profit from enhanced opportunities to bring new products to market. But attracting industry‐sponsored clinical trials was also an end in itself – a way of growing the life science innovation sector, both within and outside the NHS, to produce ‘More jobs’ and ‘Increased taxation revenue’ (Department of Trade and Industry et al. 2003, 11). The result, the government promised, would be both ‘Improved national health, through improved clinical performance and early access to innovative medicine’ and ‘Increased national wealth: enhanced Gross Domestic Product by maintaining and supporting a high growth, high margin, high value‐added, knowledge‐based industry’ (Department of Trade and Industry et al. 2003, 6). The replacement of the New Labour government by a Conservative‐Liberal Democrat coalition in 2010 did nothing to dilute the health and wealth agenda, as the Health and Social Care Act of 2012 enshrined clinical research as one of the core responsibilities of the NHS (Health and Social Care Act 2012; Faulkner‐Gurstein and Wyatt 2023).

Genomics, with its promise of research‐ and technology‐led medical revolution, fitted comfortably into the evolving policy agenda. Leading pharmaceutical innovators, including UK‐based companies like SmithKline Beecham and Astra Zeneca, were already looking to genomics as an aid to identifying druggable targets and developing targeted drugs. Their interests were well represented in policy circles, where they helped drive the creation of basic genomic research facilities like UK Biobank (House of Lords Select Committee on Science and Technology 1999). Measures to enhance clinical genetics capacity within the NHS, including upgrading clinical genetics laboratories and establishing the UK Genetic Testing Network to validate and oversee the introduction of novel tests, were also intended to advance the innovation agenda, initially by facilitating the translation of the anticipated new genetic knowledge into medical practice (Department of Health 2003, 2008; House of Lords Science and Technology Committee 2009). As the revolutionary expectations vested in GWAS and other pre‐clinical approaches began to appear increasingly ill‐founded, the policy emphasis shifted to rest instead on the NHS as the most promising site for taking forward the business of genomic innovation (Department of Health 2012).

Business is the apposite word here. Much of the impetus to develop genomics within the NHS came first from the Department of Trade and Industry and then, after the election of 2010, from the Department for Business, Innovation and Skills, which partnered with the Department of Health to publish a new Strategy for UK Life Sciences in 2011 (Department for Business, Innovation, and Skills and Office for Life Sciences 2011) and a much‐trumpeted update a year later (HM Government 2012a, 2012b). The latter document, in particular, placed clinical genomics at the centre of plans to build a thriving UK life sciences industry, as summed up in the Prime Minister's foreword: ‘Genetic science has the potential to transform healthcare systems around the world, and support the emergence of British companies creating new jobs and revenues for the UK. My ambition is nothing less than for the UK to become the world leader in this emerging industrial sector’ (HM Government 2012b, 2).

The establishment and roll‐out of the 100KGP from 2013 onwards was not just a major step towards realising that ambition. It also marked an evolution in how policy makers envisaged the role of the NHS. Enhancing the UK's ability to attract commercial clinical trials, especially in oncology, remained an important goal (Davies 2017, 9). But the architects of the 100KGP wanted to move beyond simply providing clinical services and facilities for external pharmaceutical companies. They sought now to embed commercially driven biomedical innovation much more closely into the structures and practices of the NHS itself. This was apparent in the decision to vest responsibility for delivering the 100KGP in a limited‐liability company, Genomics England, wholly owned by the Department of Health. Incorporation as a company gave Genomics England much greater freedom to ‘manage contracts for specialist UK‐based companies, universities and hospitals to supply services on sequencing, data linkage and analysis’ than would have been possible within existing NHS commissioning arrangements, as well as ‘the independence and clout to drive innovation across systems and healthcare economies’ (Department of Health and Social Care 2013).

Genomics England quickly became a nexus of commercial partnerships, especially around sequencing and data analysis. The sequencing giant Illumina was at the core of Genomic England's activities from early on, signing a £78 million deal to provide whole genome sequencing in August 2014 and, in turn, investing around £160 million into the enterprise. Further deals followed, including to provide bioinformatic services (Genomics England 2014, 2016a). Grants were awarded to smaller companies, including Congenica and Oxford Gene Technology, to support product development, particularly for data analysis and clinical interpretation software (Genomics England 2015b). Partnering with business was also expected to attune NHS staff to commercial expectations and ways of working. This was the express aim of the GENE Consortium, which ran from 2015 to 2018, bringing major pharmaceutical and biotech companies into collaboration with NHS clinicians and researchers ‘to identify the most effective and secure way of bringing industry expertise into the 100,000 Genomes Project’ and ‘making the most of their unique expertise in developing new diagnostics and treatments’ (Genomics England 2015a). In Genomics England's own assessment, the Consortium ‘allowed industry to provide advice and feedback, which has helped to steer the direction of the project’, in ways that maximised ‘the economic—as well as health benefits that genomic medicine can bring to the UK’ (Genomics England 2018b).

By 2017, the Chief Medical Officer for England – herself a longstanding champion of the idea that the NHS should be cultivated for economic growth – was promising that, by making genomic medicine a core feature of health care, ‘Our wider society will benefit not only from better health, but also by attracting new investment, creating jobs in research and the NHS, building a competitive environment that attracts world‐leading researchers and clinicians, creating a genomic literate workforce, and offering cost‐effective treatments sooner to those patients who can benefit the most’ (Davies 2017, 6). The fact that she left treatment to the end of this list of benefits might be taken as indicative of her priorities: genomic advances in diagnosis and therapy might bring relief to certain patients, but ‘the country as a whole’ could expect to benefit from economic ‘“spillovers” through the stimulation of investment in related industries’ (16). By the time these words were published, plans were already under way to extend the services and systems established under the 100KGP into an NHS‐wide Genomic Medicine Service. NHS England Board papers make clear that the senior management shared the Chief Medical Officer's vision, endorsing the ‘significant benefits for research and development that can be leveraged on behalf of the NHS, taxpayers and the wider economy. The NHS as the single biggest integrated healthcare system in the world is in a unique position … through the forthcoming life sciences industrial strategy, [to] demonstrate the nation's competitive advantage in enhancing understanding of diseases, and developing products for earlier detection and treatment’ (NHS England 2017, 3). The new service was announced to the public in summer 2018 and began rolling out in October of that year (Genomics England 2018a).

Subsequent policy developments have continued along the same trajectory, building additional capacity to support clinical trials and working through Genomics England to attract further large‐scale public–private research initiatives. Indeed, this is the primary rationale for the government's willingness to pay elevated prices for innovative medicines: the renewal of the Cancer Drugs Fund in 2016 was billed as ‘A new deal for patients, taxpayers and industry’ (NHS England 2016), while NICE's recent ‘Strategic principles for rare diseases’ do not even mention patients, declaring only the agency's aim ‘to create an attractive environment that stimulates innovation for global researchers, developers and pharmaceutical companies’ (National Institute for Health and Care Excellence 2024). Meanwhile, the scope of the envisaged innovations has grown to include other emerging big data technologies, particularly artificial intelligence and digital imaging, as well as renewed interest in the commercial possibilities of polygenic risk analysis (NHS 2019; HM Government 2020; Department of Health and Social Care 2022b). The current Labour government's vision of economic growth through investment in artificial intelligence, including in the health sector, is only the latest evolution of this agenda. The economic promise of genomics, expanded now to include other forms of data‐driven biomedical innovation, continues to shape policy. In so doing, it drives the continuing reconfiguration of the NHS as an innovation platform, further blurring the distinction between care and research and between public health care and commercial endeavour. In that respect, genomic medicine is already fulfilling its economic promise to the extent that it has helped to foster a speculative economy of biomedical innovation, in which the expansion and capitalisation of the sector counts as much as the delivery of any specific end products or applications, and even the escalating price of new medicines can be seen not as a cost but as an investment.

## 5 Human Value

It is important to think about what all this might mean for patients. On the one hand, the accelerating pace of genomic innovation means that growing numbers of patients have been able to avail themselves of remarkable new diagnostic and, to a lesser extent, therapeutic opportunities. On the other hand, as the NHS itself has been reconfigured to support the business of biomedical innovation, patients and their families have increasingly been cast in a different role, not just as recipients of care but as experimental subjects and data sources. In effect, patients and their personal health data have become an economic resource – one of the key assets that make the NHS appear such a promising site for the pursuit of wealth as well as health. This framing is apparent in policy documents going back at least as far as the beginnings of the health and wealth agenda. The ability to access large numbers of patients was a crucial factor in government plans to make the United Kingdom a favoured location to conduct clinical research: the ‘largely unified structure’ of the NHS meant that ‘clinical trials can span a number of hospitals, or cross primary and secondary care’ (HM Government 2005, 2). But as the Office for Life Sciences put it, ‘The UK can do much more to harness the opportunity that exists in the NHS’, particularly by making it easier ‘to involve patients in trials and early access schemes for the treatment of chronic diseases, such as cancer’ (Department for Business, Innovation, and Skills and Office for Life Sciences 2011, 5)

One way of achieving this was by making research ethics procedures more researcher friendly. Access to personal medical data was seen as particularly important in this regard. In 2003, the Academy of Medical Sciences called for ‘a National Ethical Code for informed consent relating to patient data’ in order to ‘simplify access to patient records for ethically approved research projects that would have no direct impact on the individual patient’ (Academy of Medical Sciences 2003, 10). Three years later, under the heading ‘Bureaucracy busting’, the Research and Development Directorate of the Department of Health declared its aim ‘to promote a regulatory and governance environment that both facilitates high‐quality research and protects the rights, dignity and safety of those who agree to take part. We will promote research governance processes that are proportionate to risk’ (Department of Health and Social Care 2006, 13). As clinical genomics came to occupy an increasingly prominent place in government plans for biomedical innovation, the scope of what participants consented to was broadened, from allowing their data to be used for specified purposes, to permitting any research use of their data, to consenting to be recontacted for additional studies (Department of Health and Social Care 2022b)

Loosening the ethical restrictions on recruiting patients and accessing their data did not mean that patients would automatically be more inclined to participate in research, however. Participation is rarely without cost to patients, who do not usually shoulder that cost without reason. They generally do so in return for a promise – in particular, the expectation that their participation will lead to good, be it for themselves or for others. Genomic medicine policy was full of such promises. Benefit to research participants themselves was a recurring theme. Clinical trials were represented as a way for patients to secure early access to promising new treatments – and even if that promise proved unfounded, policy makers argued, trial participants would still benefit, because ‘protocol‐driven care improves both patient outcomes and the skills of healthcare professionals’ (Department of Trade and Industry et al. 2003, 7). The introduction of new genomic technologies into clinical research offered other kinds of patient benefits: ‘Some participating patients will benefit because a conclusive diagnosis can be reached for a rare and inherited disease more quickly, or because a treatment for cancer can be targeted at the particular genetic change that is present in the cancer’, declared NHS leaders, as provisions for whole genome sequencing were put in place and patients began to be recruited (NHS England 2014). Self‐interest could not be the only motivation, though. Research outcomes are by their nature unpredictable, new treatments are often ineffective or even harmful and advanced technologies do not always bring an end to diagnostic or therapeutic uncertainty. In such instances, policy makers appealed to a greater good, beyond personal benefit: ‘for a number of patients, the benefit will be in the improvement in our knowledge of the influence of genetics on disease … [and] how other people can be helped with similar diseases in the future’ (NHS England 2014; cf. Petersen 2005). Appeals to altruism were in turn embedded in ideas of a more general ‘social contract’, in which patients and families would ‘agree to use of data within a clinical and confidential framework’, in the expectation that it would ‘benefit the patient's own care, their family and that of others’ (Davies 2017, 16; see also Academy of Medical Sciences 2015).

Policy makers were unsure whether patients would be swayed by such promises, however. Consequently, a degree of persuasion might be required. ‘Key to advancing genomic medicine will be *helping patients to understand* that by agreeing to use of data about their illness, they bring direct benefits to themselves’, warned the Chief Medical Officer in setting out her plans for a Genomic Medical Service (Davies 2017, 5, my emphasis). She and others were particularly worried about attitudes towards the involvement of commercial interests. Early soundings had made it clear that while potential research participants were often more than willing to share their time and data with public organisations such as the NHS – the embodiment, for many, of the kind of social contract that policy makers alluded to – they often baulked at the idea of commercial companies profiting from their data. The 100KGP therefore included a ‘public communication and engagement plan’ aimed at ‘establish[ing] the correct approach within the NHS to capitalise on the creation of a health care system able to generate and use large amounts of genetic data to improve the health of patients’ (HM Government 2012a, 49). It concluded, among other things, that ‘industry's role in developing new drugs and treatments and how they work with public institutions and the NHS is not widely understood. More needs to be done to explain their role’ (Genomics England 2016b, 22; see also Ipsos MORI 2016). Much of the work of explanation and persuasion might be undertaken by organisations with particular expertise in public engagement and communications. But some champions of genomic medicine envisaged that medical practitioners, too, could play a part in steering patients in particular directions: ‘it may be that the challenges presented by the uncertainties and open‐endedness of genomic medicine require a rethinking of some aspects of the role of non‐directedness in the doctor‐patient relationship’ (Davies 2017, ch. 16, p. 6).

The suggestion that the principle of non‐directive clinical practice might need rethinking should give us pause. The instance just quoted comes towards the end of a lengthy report on the current state and future promise of genomic medicine and is discussed only briefly. But the fact that it is mentioned at all, especially in the context of clinical genetics, signals a potentially historic shift in attitudes towards patients. Non‐directiveness has been fundamentally important for clinical geneticists since the 1970s. It gave practical expression to their repudiation of the evils of eugenics and their rejection of the discriminatory and prejudicial eugenic policies and practices to which many geneticists had contributed during the first half of the 20th century. By seeking, as far as possible, to counsel patients and families in ways that enabled them to make their own informed decisions, clinical geneticists sought to ensure that their specialty would never again collude in the kind of social engineering that characterised eugenics. I do not wish in the present essay to suggest that the current challenge to clinical non‐directiveness implies that contemporary genomic medicine somehow perpetuates or reproduces aspects of eugenics (though the question is moot: see e.g. Duster 2003). But I do worry that the idea that doctors should be encouraged to steer their patients towards research participation, along with the loosening of consent procedures and the introduction of measures to persuade patients of the value of industry involvement in genomic research, reflects a willingness to prioritise a particular kind of economic promise over what patients might themselves hope for from the healthcare system.

The pursuit of economic growth through biomedical innovation is not necessarily at odds with the pursuit of medical improvements in the lives of patients and their families. Indeed, the promissory rhetoric that has long attended the development of genomic medicine routinely assumes that the one helps drive the other. But such assumptions tend to elide patients’ own concerns about the direction and purpose of research and their role in it. Patients are remarkably generous in their willingness to contribute to the advancement of medical research, if they believe it serves a wider public good. But how they understand that good, and how they orient themselves towards it, is profoundly shaped by context (Stemerding and Nelis 2006; Tutton and Prainsack 2011). The fact that, in the United Kingdom, so much genomic research is conducted within a highly regarded National Health Service goes a long way to reassure participants that the public good is indeed the central aim (e.g. Dheensa et al. 2019). The involvement of commercial organisations, on the other hand, is a major deterrent. Patients object to the idea that their contributions to public research might be appropriated to generate private profit. More vaguely, they worry that the incursion of private interests might mark a step towards the commercialisation of the NHS and the extraction of private value at the expense of a respected public institution.

The analysis I have set out in this article suggests that patients’ fears are not entirely ill‐founded. As we have seen, the medical benefits delivered to date through the pursuit of genomic medicine are markedly disproportionate to the investments – financial, organisational and personal – that have made them possible. Given the present trajectory of the field, moreover, the cost–benefit imbalance only looks likely to get worse, forcing the NHS to make increasingly difficult choices as it struggles to fund the expensive new drugs and treatments that the current industry‐oriented biomedical innovation system aims to deliver. In that respect, patients’ apprehensions are broadly in line with the way that genomic medicine is actually developing.

Conflict between economic and medical interests may not be inevitable, but in its current configuration, genomic medicine embodies a real – if largely unacknowledged – tension between patients’ and policy makers’ visions. That tension is often obscured by vagueness and ambiguity in the language used, especially in such evocative terms as ‘the public good’ (e.g. Metcalf and Sadowski 2024; Sterckx et al. 2018). And it does not exist solely at the level of policy. Clinicians experience it in their daily practice, as they navigate between the imperatives of research on the one hand and the delivery of care on the other (Dheensa et al. 2018; Nuffield Council on Bioethics 2015). The idea that, in addition, clinicians should now abandon their commitment to non‐directive counselling, and should instead try to steer their patients to participate in research, presumes that clinicians and patients alike should swallow their concerns about commercialisation and defer to the interests of policy makers and other cheerleaders for biomedical innovation.

Genomic medicine is rich with promises – as much so now as in the heady days that followed the completion of the Human Genome Project. Promises have been instrumental in driving and shaping genomic medicine as it now exists, and they continue to inform how it develops towards the future. But as we have seen, there are different kinds of promise in play that appeal to different constituencies with different interests and different values and that are not always consistent or compatible with one another. In that regard, much of the promissory discourse surrounding genomic medicine can be seen as complementing other initiatives, including efforts to educate patients about the benefits of research participation and calls to abandon the principle of non‐directiveness, which aim to overcome participants’ worries about the ends of commercially oriented biomedical innovation. Given the issues at stake – the need for effective ways of dealing with dire diseases and disorders, but also the question of the direction and sustainability of genomic health care – it is surely high‐handed and undemocratic to foreclose in this way on the range of relevant interests, opinions and judgements. In particular, we ought to heed rather than over‐ride the concerns of patients, who are at once the most generous and the most vulnerable of the constituencies involved in the development of genomic medicine. Given everything that patients and the tax‐paying UK public have contributed to that development, it is imperative that we think carefully about what we promise them, if only to ensure that we do not exploit their generosity to advance causes they would not approve.

## Author Contributions

The conception, research and writing of this article were entirely the author's own work.

## Data Availability

The data on which this study is based all derive from published works. These sources are all fully referenced in the text of the article.

## References

[ahg70011-bib-0001] Academy of Medical Sciences . 2003. “Strengthening Clinical Research: A Report From the Academy of Medical Sciences.” https://acmedsci.ac.uk/file‐download/34704‐pscr.pdf.

[ahg70011-bib-0002] Academy of Medical Sciences . 2015. “A New Social Contract for Medical Innovation.” https://acmedsci.ac.uk/more/news/a‐new‐social‐contract‐for‐medical‐innovation.

[ahg70011-bib-0003] Atkinson, P. , S. Sheard , and T. Walley . 2019. “‘All the Stars Were Aligned?’ The Origins of England's National Institute for Health Research.” Health Research Policy and Systems 17, no. 1: 95. 10.1186/s12961-019-0491-5. 31801552 PMC6894247

[ahg70011-bib-0004] Borup, M. , N. Brown , K. Konrad , and H. van Lente . 2006. “The Sociology of Expectations in Science and Technology.” Technology Analysis & Strategic Management 3/4: 285–298.

[ahg70011-bib-0005] Brown, N. , and M. Michael . 2003. “A Sociology of Expectations: Retrospecting Prospects and Prospecting Retrospects.” Technology Analysis & Strategic Management 15, no. 1: 3–18.

[ahg70011-bib-0006] Cambrosio, A. , P. Keating , E. Vignola‐Gagné , S. Besle , and P. Bourret . 2018. “Extending Experimentation: Oncology's Fading Boundary Between Research and Care.” New Genetics and Society 37, no. 3: 207–226. 10.1080/14636778.2018.1487281.

[ahg70011-bib-0007] Cook‐Deegan, R. 1994. The Gene Wars: Science, Politics and the Human Genome. W.W. Norton & Company.

[ahg70011-bib-0008] Cooksey, D. 2006. “A Review of UK Health Research Funding.” https://assets.publishing.service.gov.uk/media/5a7c49bc40f0b6321db382e2/0118404881.pdf. 10.1136/bmj.39059.444120.80 PMC1702444 17170394

[ahg70011-bib-0009] Cooper, M. , and C. Waldby . 2014. Clinical Labor: Tissue Donors and Research Subjects in the Global Bioeconomy. Duke University Press.

[ahg70011-bib-0010] Davies, S. C. 2017. Annual Report of the Chief Medical Officer 2016, Generation Genome. Department of Health.

[ahg70011-bib-0011] Day, S. , R. C. Coombes , L. McGrath‐Lone , C. Schoenborn , and H. Ward . 2016. “Stratified, Precision or Personalised Medicine? Cancer Services in the “Real World” of a London Hospital.” Sociology of Health and Illness 39, no. 1: 143–158. 27460935 10.1111/1467-9566.12457

[ahg70011-bib-0012] Day, S. , W. Viney , J. Bruton , and H. Ward . 2021. “Past‐Futures in Experimental Care: Breast Cancer and HIV Medicine.” New Genetics and Society 40, no. 4: 449–472. 10.1080/14636778.2020.1861542.

[ahg70011-bib-0013] Department for Business, Innovation and Skills, and Office for Life Sciences . 2011. “Strategy for UK Life Sciences.” https://www.gov.uk/government/publications/uk‐life‐sciences‐strategy.

[ahg70011-bib-0014] Department of Health and Social Care . 2006. “Best Research for Best Health: A New National Health Research Strategy.” https://www.gov.uk/government/publications/best‐research‐for‐best‐health‐a‐new‐national‐health‐research‐strategy.

[ahg70011-bib-0015] Department of Health and Social Care . 2013. “Press Release: DNA Mapping to Better Understand Cancer, Rare Diseases and Infectious Diseases.” https://www.gov.uk/government/news/dna‐mapping‐to‐better‐understand‐cancer‐rare‐diseases‐and‐infectious‐diseases.

[ahg70011-bib-0016] Department of Health and Social Care . 2018. “The UK Strategy for Rare Diseases: Rare Diseases Implementation Plan for England.” https://assets.publishing.service.gov.uk/media/5a783b32ed915d07d35b0c86/UK_strategy_for_rare_diseases_‐_implementation_plan_for_England.pdf.

[ahg70011-bib-0017] Department of Health and Social Care . 2021. “The UK Rare Diseases Framework.” https://www.gov.uk/government/publications/uk‐rare‐diseases‐framework/the‐uk‐rare‐diseases‐framework.

[ahg70011-bib-0018] Department of Health and Social Care . 2022a. “England Rare Diseases Action Plan 2022.” Published February 28. https://www.gov.uk/government/publications/england‐rare‐diseases‐action‐plan‐2022/england‐rare‐diseases‐action‐plan‐2022.

[ahg70011-bib-0019] Department of Health and Social Care . 2022b. “Genome UK: 2022 to 2025 Implementation Plan for England.” Published December 13. https://www.gov.uk/government/publications/genome‐uk‐2022‐to‐2025‐implementation‐plan‐for‐england/genome‐uk‐2022‐to‐2025‐implementation‐plan‐for‐england.

[ahg70011-bib-0020] Department of Health and Social Care . 2022c. “Press Release: Patients to Have Earlier Access to Cutting‐Edge Treatments on NHS.” Published June 7. https://www.gov.uk/government/news/patients‐to‐have‐earlier‐access‐to‐cutting‐edge‐treatments‐on‐nhs.

[ahg70011-bib-0021] Department of Health . 2003. Our Inheritance, Our Future: Realising the Potential of Genetics in the NHS. Department of Health.

[ahg70011-bib-0022] Department of Health . 2008. “Our Inheritance, Our Future: Realising the Potential of Genetics in the NHS.” http://data.parliament.uk/DepositedPapers/Files/DEP2008‐1084/DEP2008‐1084.pdf.

[ahg70011-bib-0023] Department of Health . 2012. “Building on Our Inheritance: Genomic Technology in Healthcare.” https://www.gov.uk/government/publications/genomic‐technology‐in‐healthcare‐building‐on‐our‐inheritance.

[ahg70011-bib-0024] Department of Health, and Association of the British Pharmaceutical Industry . 2001. “Pharmaceutical Industry Competitiveness Task Force, Final Report.” https://www.rieti.go.jp/jp/events/bbl/data/pictf.pdf.

[ahg70011-bib-0025] Department of Trade and Industry, Bioscience Innovation and Growth Team, Department of Health, and BioIndustry Association . 2003. Bioscience 2015: Improving National Health, Increasing National Wealth: *Executive Summary* . Department of Trade and Industry.

[ahg70011-bib-0026] Dheensa, S. , G. Samuel , A. M. Lucassen , and B. Farsides . 2018. “Towards a National Genomics Medicine Service: The Challenges Facing Clinical‐Research Hybrid Practices and the Case of the 100 000 Genomes Project.” Journal of Medical Ethics 44, no. 6: 397–403. 29496751 10.1136/medethics-2017-104588 PMC5992369

[ahg70011-bib-0027] Dheensa, S. , A. Lucassen , and A. Fenwick . 2019. “Fostering Trust in Healthcare: Participants' Experiences, Views, and Concerns About the 100,000 Genomes Project.” European Journal of Medical Genetics 62, no. 5: 335–341. 10.1016/j.ejmg.2018.11.024. 30503854

[ahg70011-bib-0028] Duster, T. 2003. Backdoor to Eugenics. Routledge.

[ahg70011-bib-0029] Faulkner‐Gurstein, R. , and D. Wyatt . 2023. “Platform NHS: Reconfiguring a Public Service in the Age of Digital Capitalism.” Science, Technology, & Human Values 48, no. 4: 888–908. https://doi.org.eux.idm.oclc.org/10.1177/01622439211055697.

[ahg70011-bib-0030] Firth, H. V. , and C. F. Wright . 2011. “The Deciphering Developmental Disorders (DDD) Study.” Developmental Medicine and Child Neurology 53, no. 8: 702–703. 21679367 10.1111/j.1469-8749.2011.04032.x

[ahg70011-bib-0031] García‐Sancho, M. , R. Leng , G. Viry , M. Wong , N. Vermeulen , and J. Lowe . 2022. “The Human Genome Project as a Singular Episode in the History of Genomics.” Historical Studies in the Natural Sciences 52, no. 3: 320–360. 10.1525/hsns.2022.52.3.320.

[ahg70011-bib-0032] Genomics England . 2014. “UK to Become World Number One in DNA Testing With Plan to Revolutionise Fight Against Cancer and Rare Diseases.” Published August 1. https://www.genomicsengland.co.uk/news/uk‐becomes‐number‐one‐in‐dna‐testing.

[ahg70011-bib-0033] Genomics England . 2015a. “Clinicians, Researchers and Industry Collaborate With the 100,000 Genomes Project.” Published March 26. https://www.genomicsengland.co.uk/news/collaborations‐100000‐genomes‐project.

[ahg70011-bib-0034] Genomics England . 2015b. “Genomics England Announces Successful Companies in Small Business Research Initiative (SBRI) for a Share of £8 Million.” Published March 11. https://www.genomicsengland.co.uk/news/genomics‐england‐announces‐successful‐companies‐in‐sbri.

[ahg70011-bib-0035] Genomics England . 2016a. “Genomics England Enters Bioinformatics Partnership With Illumina.” Published February 11. https://www.genomicsengland.co.uk/news/bioinformatics‐partnership‐with‐illumina.

[ahg70011-bib-0036] Genomics England . 2016b. “The Genomics Conversation—An Overview.” https://www.genomicsengland.co.uk/assets/images/News‐and‐Events/News‐Articles‐Images/The‐Genomics‐Conversation‐Overview‐FINAL2‐081216.pdf.

[ahg70011-bib-0037] Genomics England . 2018a. “As the NHS Celebrates 70 Years Genomics England Sequences Its 70,000th Genome.” Published July 4. https://www.genomicsengland.co.uk/news/nhs‐celebrates‐70‐years‐70000th‐genome.

[ahg70011-bib-0038] Genomics England . 2018b. “GENE Consortium Legacy Steers Industry Cooperation at Genomics England.” Published July 28. https://www.genomicsengland.co.uk/news/gene‐consortium‐legacy.

[ahg70011-bib-0040] Health and Social Care Act 2012 c. 7. https://www.legislation.gov.uk/ukpga/2012/7/.

[ahg70011-bib-0041] Hedgecoe, A. 2004. The Politics of Personalised Medicine: Pharmacogenetics in the Clinic. Cambridge University Press.

[ahg70011-bib-0042] Hedgecoe, A. , and P. Martin . 2003. “The Drugs Don't Work: Expectations and the Shaping of Pharmacogenetics.” Social Studies of Science 33, no. 2: 327–364. 14621671 10.1177/03063127030333002

[ahg70011-bib-0043] Heeney, C. 2021. “Problems and Promises: How to Tell the Story of a Genome Wide Association Study?” Studies in the History and Philosophy of Science Part A 89: 1–10. 10.1016/j.shpsa.2021.06.003. 34284196

[ahg70011-bib-0044] Hingorani, A. D. , J. Gratton , C. Finan , et al. 2023. “Performance of Polygenic Risk Scores in Screening, Prediction, and Risk Stratification: Secondary Analysis of Data in the Polygenic Score Catalog.” BMJ Medicine 2: e000554. 10.1136/bmjmed-2023-000554. 37859783 PMC10582890

[ahg70011-bib-0045] HM Government . 2005. “Government Response to the Health Committee's Report on the Influence of the Pharmaceutical Industry.” https://assets.publishing.service.gov.uk/media/5a7b90daed915d414762135a/6655.pdf.

[ahg70011-bib-0046] HM Government . 2012a. “DNA Tests to Revolutionise Fight Against Cancer and Help 100,000 NHS Patients.” https://www.gov.uk/government/news/dna‐tests‐to‐revolutionise‐fight‐against‐cancer‐and‐help‐100000‐nhs‐patients.

[ahg70011-bib-0047] HM Government . 2012b. “Strategy for UK Life Sciences: One Year On.” https://www.gov.uk/government/publications/strategy‐for‐uk‐life‐sciences‐one‐year‐on.

[ahg70011-bib-0048] HM Government . 2020. “Genome UK: The Future of Healthcare.” https://www.gov.uk/government/publications/genome‐uk‐the‐future‐of‐healthcare.

[ahg70011-bib-0049] HM Government . 2021. “Genome UK: 2021 to 2022 Implementation Plan.” https://www.gov.uk/government/publications/genome‐uk‐2021‐to‐2022‐implementation‐plan.

[ahg70011-bib-0050] House of Lords, Science and Technology Committee . 2009. Second Report: Genomic Medicine, Volume 1: Report, HL 107‐I. The Stationery Office. https://publications.parliament.uk/pa/ld200809/ldselect/ldsctech/ldsctech.htm.

[ahg70011-bib-0051] House of Lords, Select Committee on Science and Technology . 1999. “Human Genetic Databases: Second Report, Meeting with Health and Science Ministers, HL 11.” https://publications.parliament.uk/pa/ld199900/ldselect/ldsctech/11/1102.htm.

[ahg70011-bib-0052] International HapMap Consortium . 2003. “The International HapMap Project.” Nature 426: 789–796. 14685227 10.1038/nature02168

[ahg70011-bib-0053] International Human Genome Sequencing Consortium . 2001. “Initial Sequencing and Analysis of the Human Genome.” Nature 409, no. 6822: 860–921. 10.1038/35057062. 11237011

[ahg70011-bib-0054] International Human Genome Sequencing Consortium . 2004. “Finishing the Euchromatic Sequence of the Human Genome.” Nature 431, no. 7011: 931–945. 10.1038/nature03001. 15496913

[ahg70011-bib-0055] Ipsos MORI . 2016. “The One‐Way Mirror: Public Attitudes to Commercial Access to Health Data.” https://wellcome.org/sites/default/files/public‐attitudes‐to‐commercial‐access‐to‐health‐data‐wellcome‐mar16.pdf.

[ahg70011-bib-0056] Kerr, A. , C. K. Chekar , E. Ross , J. E. Swallow , and S. Cunningham‐Burley . 2021. Personalised Cancer Medicine: Future Crafting in the Genomic Era. Manchester University Press. 33555770

[ahg70011-bib-0057] Lander, E. S. , and N. J. Schork . 1994. “Genetic Dissection of Complex Traits.” Science 265: 2037–2048. 8091226 10.1126/science.8091226

[ahg70011-bib-0058] Metcalf, K. , and J. Sadowski . 2024. “Asserting the Public Interest in Health Data: On the Ethics of Data Governance for Biobanks and Insurers.” Big Data & Society 11, no. 4. 10.1177/20539517241290215.

[ahg70011-bib-0059] Mitchell, R. 2010. “National Biobanks: Clinical Labor, Risk Production, and the Creation of Biovalue.” Science, Technology, & Human Values 35, no. 3: 330–355. 10.1177/0162243909340267. PMC2879701 20526462

[ahg70011-bib-0060] National Human Genome Research Institute . 2000. “Human Genome Project and SNP Consortium Announce Collaboration to Identify New Genetic Markers for Disease.” www.genome.gov/10001456.

[ahg70011-bib-0061] National Human Genome Research Institute . 2002. “International Consortium Launches Genetic Variation Mapping Project.” www.genome.gov/10005336.

[ahg70011-bib-0062] National Institute for Health and Care Excellence . 2024. “Strategic Principles for Rare Diseases.” https://www.nice.org.uk/process/pmg46/resources/nice‐strategic‐principles‐a‐complementary‐approach‐to‐public‐health‐social‐care‐and‐rare‐disease‐topics‐13430355949/chapter/strategic‐principles‐for‐rare‐diseases.

[ahg70011-bib-0063] National Institute of Health and Care Excellence . 2022. “NICE Health Technology Evaluation Topic Selection: The Manual. 7.1 Highly Specialised Technologies.” https://www.nice.org.uk/process/pmg37/chapter/highly‐specialised‐technologies.

[ahg70011-bib-0064] NHS England . 2014. “NHS Set to Deliver World‐Leading Genomics Project in Fight Against Cancer and Rare Diseases.” https://www.england.nhs.uk/2014/12/genomics‐project/.

[ahg70011-bib-0065] NHS England . 2016. “Appraisal and Funding of Cancer Drugs from July 2016 (Including the New Cancer Drugs Fund).” https://www.england.nhs.uk/wp‐content/uploads/2024/04/appraisal‐and‐funding‐of‐cancer‐drugs‐from‐July‐2016.pdf.

[ahg70011-bib-0066] NHS England . 2017. “Board Paper PB.30.03.2017/06, Creating a Genomic Medicine Service to Lay the Foundations to Deliver Personalised Interventions and Treatments.” https://www.england.nhs.uk/publication/board‐papers‐30‐march‐2017/.

[ahg70011-bib-0067] NHS . 2019. “The NHS Long Term Plan, Version 1.2 With Corrections.” https://www.longtermplan.nhs.uk/wp‐content/uploads/2019/08/nhs‐long‐term‐plan‐version‐1.2.pdf.

[ahg70011-bib-0068] Nuffield Council on Bioethics . 2015. “The Collection, Linking and Use of Data in Biomedical Research.” https://www.nuffieldbioethics.org/publication/the‐collection‐linking‐and‐use‐of‐data‐in‐biomedical‐research‐and‐health‐care‐ethical‐issues/.

[ahg70011-bib-0069] Nurk, S. , Koren , S. , A. Rhie , et al. 2022. “The Complete Sequence of a Human Genome.” Science 376, no. 6588: 44–53. 10.1126/science.abj6987. 35357919 PMC9186530

[ahg70011-bib-0070] Ochoa, D. , M. Karim , M. Ghoussaini , D. G. Hulcoop , E. M. McDonagh , and I. Dunham . 2022. “Human Genetics Evidence Supports Two‐Thirds of the 2021 FDA‐Approved Drugs.” Nature Reviews. Drug Discovery 21, no. 8: 551–551. 10.1038/d41573-022-00120-3. 35804044

[ahg70011-bib-0071] Pennisi, E. 2007. “Breakthrough of the Year: Human Genetic Variation.” Science 318, no. 5858: 1842–1843. 18096770 10.1126/science.318.5858.1842

[ahg70011-bib-0072] Petersen, A. 2005. “Securing Our Genetic Health: Engendering Trust in UK Biobank.” Sociology of Health & Illness 27, no. 2: 271–292. 10.1111/j.1467-9566.2005.00442.x. 15787778

[ahg70011-bib-0073] Prasad, V. , K. de Jesús , and S. Mailankody . 2017. “The High Price of Anticancer Drugs: Origins, Implications, Barriers, Solutions.” Nature Reviews Clinical Oncology 17, no. 14: 381–390. 10.1038/nrclinonc.2017.31. 28290490

[ahg70011-bib-0074] Rare Disease Research Landscape Steering Group . 2023. “Rare Diseases Research Landscape Project Report [Version 1; Not Peer Reviewed].” NIHR Open Research. https://openresearch.nihr.ac.uk/documents/3‐45.

[ahg70011-bib-0075] Reardon, J. 2017. The Postgenomic Condition: Ethics, Justice, and Knowledge After the Genome. Chicago University Press.

[ahg70011-bib-0076] Rusina, P. V. , M. J. Falaguera , J. M. R. Romero , E. M. McDonagh , I. Dunham , and D. Ochoa . 2023. “Genetic Support for FDA‐Approved Drugs Over the Past Decade.” Nature Reviews Drug Discovery 22, no. 11: 864–864. 10.1038/d41573-023-00158-x. 37803084

[ahg70011-bib-0077] Saluja, R. , V. S. Arciero , S. Cheng , et al. 2018. “Examining Trends in Cost and Clinical Benefit of Novel Anticancer Drugs Over Time.” Journal of Oncology Practice 14: e280–e294. 10.1200/JOP.17.00058. 29601250

[ahg70011-bib-0078] Samuel, G. N. , and B. Farsides . 2017. “The UK's 100,000 Genomes Project: Manifesting Policymakers' Expectations.” New Genetics and Society 36, no. 4: 336–353. 10.1080/14636778.2017.1370671. 29238265 PMC5706982

[ahg70011-bib-0079] Schnog, J. B. , M. J. Samson , R. O. B. Gans , and A. J. Duits . 2021. “An Urgent Call to Raise the Bar in Oncology.” British Journal of Cancer 125: 1477–1485. 34400802 10.1038/s41416-021-01495-7 PMC8365561

[ahg70011-bib-0080] Shendure, J. , G. M. Findlay , and M. W. Snyder . 2019. “Genomic Medicine—Progress, Pitfalls, and Promise.” Cell 177, no. 1: 45–57. 10.1016/j.cell.2019.02.003. 30901547 PMC6531313

[ahg70011-bib-0081] Sosinsky, A. , J. Ambrose , W. Cross et al. 2024. “Insights for Precision Oncology From the Integration of Genomic and Clinical Data of 13,880 Tumors From the 100,000 Genomes Cancer Programme.” Nature Medicine 30, no. 1: 279–289. 10.1038/s41591-023-02682-0. PMC10803271 38200255

[ahg70011-bib-0082] Stemerding, D. , and A. Nelis . 2006. “Cancer Genetics and Its ‘Different Faces of Autonomy’.” New Genetics and Society 25, no. 1: 1–19. 17312627 10.1080/14636770600603329

[ahg70011-bib-0083] Sterckx, S. , S. Dheensa , and J. Cockbain . 2018. “Presuming the Promotion of the Common Good by Large‐Scale Health Research: The Cases of care.data 2.0 and the 100,000 Genomes Project in the UK.” In Personalised Medicine, Individual Choice and the Common Good, edited by B. van Beers D. Dickenson , and S. Sterckx , 155–182. Cambridge University Press. 10.1017/9781108590600.008.

[ahg70011-bib-0084] Sturdy, S. 2020. “Finding the Global in the Local: Constructing Population in the Search for Disease Genes.” In Global Health and the New World Order: Historical and Anthropological Approaches of a Transition, edited by J.‐P. Gaudillière , C. Beaudevin , C. Gradmann , A. M. Lovell , and L. Pordié , 154–182. Manchester University Press. 33226758

[ahg70011-bib-0085] Sturdy, S. 2021. “Local Mutations: On the Tentative Beginnings of Molecular Oncology in Britain 1980–2000.” New Genetics and Society 40, no. 1: 7–25. 10.1080/14636778.2021.1880887.

[ahg70011-bib-0086] Sullivan, R. 2024. “Cancer Medicines: A Private Vice for Public Benefit?” Ecancermedicalscience 18: ed131. 10.3332/ecancer.2024.ed131. 38425769 PMC10901629

[ahg70011-bib-0087] Torkamani, A. , N. E. Wineinger , and E. J. Topol . 2018. “The Personal and Clinical Utility of Polygenic Risk Scores.” Nature Reviews Genetics 19: 581–590. 10.1038/s41576-018-0018-x 29789686

[ahg70011-bib-0088] Turro, E. , W. J. Astle , K. Megy , et al. 2020. “Whole‐Genome Sequencing of Patients With Rare Diseases in a National Health System.” Nature 583, no. 7814: 96–102. 10.1038/s41586-020-2434-2. 32581362 PMC7610553

[ahg70011-bib-0089] Tutton, R. 2014. Genomics and the Reimagining of Personalized Medicine. Ashgate.

[ahg70011-bib-0090] Tutton, R. , and B. Prainsack . 2011. “Enterprising or Altruistic Selves? Making Up Research Subjects in Genetics Research.” Sociology of Health & Illness 33, no. 7: 1081–1095. 10.1111/j.1467-9566.2011.01348.x. 21507012

[ahg70011-bib-0091] UK10K Consortium . 2011. “UK10K Goals and Aims.” https://www.uk10k.org/goals.html/.

[ahg70011-bib-0092] UK10K Consortium . 2015. “The UK10K Project Identifies Rare Variants in Health and Disease.” Nature 526: 82–90. 10.1038/nature14962. 26367797 PMC4773891

[ahg70011-bib-0093] Visscher, P. M. , N. R. Wray , Q. Zhang , et al. 2017. “10 Years of GWAS Discovery: Biology, Function, and Translation.” American Journal of Human Genetics 101, no. 1: 5–22. 10.1016/j.ajhg.2017.06.005. 28686856 PMC5501872

[ahg70011-bib-0094] Wanless, D. 2002. “Securing our Future Health: Taking a Long Term View.” www.hm‐treasury.gov.uk/Consultations_and_legislation/wanless.

[ahg70011-bib-0095] Wellcome Trust Case Control Consortium . 2007. “Genome‐Wide Association Study of 14,000 Cases of Seven Common Diseases and 3,000 Shared Controls.” Nature 447: 661–678. 10.1038/nature05911. 17554300 PMC2719288

[ahg70011-bib-0096] Weymann, D. , J. Buckell , P. Fahr , et al. 2024. “Health Care Costs After Genome‐Wide Sequencing for Children With Rare Diseases in England and Canada.” JAMA Network Open 7, no. 7: e2420842. 10.1001/jamanetworkopen.2024.20842. 38985473 PMC11238031

[ahg70011-bib-0097] White House, Office of the Press Secretary . 2000. “Remarks Made by the President, Prime Minister Tony Blair of England (via Satellite), Dr. Francis Collins, Director of the National Human Genome Research Institute, and Dr. Craig Venter, President and Chief Scientific Officer, Celera Genomics Corporation, on the Completion of the First Survey of the Entire Human Genome Project.” https://www.genome.gov/10001356.

[ahg70011-bib-0098] Wigby, K. M. , D. Brockman , G. Costain , et al. 2024. “Evidence Review and Considerations for Use of First Line Genome Sequencing to Diagnose Rare Genetic Disorders.” npj Genomic Medicine 9: 15. 10.1038/s41525-024-00396-x. 38409289 PMC10897481

